# Inducible re-expression of p16 in an orthotopic mouse model of pancreatic cancer inhibits lymphangiogenesis and lymphatic metastasis

**DOI:** 10.1038/sj.bjc.6604457

**Published:** 2008-06-24

**Authors:** P Schulz, A Scholz, A Rexin, P Hauff, M Schirner, B Wiedenmann, K Detjen

**Affiliations:** 1 Department of Hepatology and Gastroenterology, Charité-Universitätsmedizin Berlin, Campus Virchow-Klinikum, Berlin 13353, Germany; 2 Global Drug Discovery, Bayer Schering Pharma AG, Berlin 13342, Germany

**Keywords:** p16^INK4a^, pancreatic cancer, orthotopic model, lymphangiogenesis, metastasis

## Abstract

Functional inactivation of the tumour suppressor protein p16^INK4a^ constitutes a key event in the multistep process of pancreatic ductal cell transformation. However, the significance of p16 inactivation for complex and tissue-specific aspects of pancreatic cancer progression, such as angiogenesis and metastasis, is less understood. Here, we inducibly re-expressed p16 *in vivo* in an orthotopic model of pancreatic cancer and examined the impact on these clinically relevant aspects of pancreatic cancer tumour biology. Consistent with previous work in subcutaneous xenograft models, we found p16 capable of reducing primary tumour growth. In addition, p16 restitution resulted in a marked reduction of tumour angiogenesis, largely accounted for by a p16-dependent inhibition of lymphangiogenesis. In excellent agreement with the antilymphangiogenic effect, re-expression of p16 almost completely prevented lymph node metastases of MiaPaca-2 pancreatic tumours. To our knowledge, this is the first report that experimentally links the tumour suppressor p16 to the process of lymphangiogenesis.

At the time of diagnosis, most patients with pancreatic cancer require systemic therapy as invasive growth and metastatic spread occur early in the course of the disease (Hezel *et al*, 2006; Chu *et al*, 2007). Unfortunately, pancreatic cancer has proven remarkably resistant to current systemic treatment modalities including chemo- and radiotherapies (Xiong *et al*, 2006), suggesting a unique combination of molecular alterations accounts for this highly unfavourable biological profile. Several molecular alterations were identified, such that pancreatic cancer is now perceived as a genetic disease characterised by the sequence of oncogenic K-ras activation, inactivation of the tumour suppressor p16^INK4a^, and inactivation of the tumour suppressors p53 and/or DPC4. Most pancreatic cancer cells harbour a combination of 3–4 of the alterations (Rozenblum *et al*, 1997). Activation of K-ras in virtually all samples of ductal pancreatic adenocarcinoma marks the initiation of the multistep transformation process, but functional inactivation of p16^INK4a^ in up to 90% of cases ranks second in frequency (Caldas *et al*, 1994; Hezel *et al*, 2006).

p16 was discovered as a tumour suppressor and an inhibitor of cyclin D-associated CDK complexes. The main biological function of p16^INK4a^ indeed involves the regulation of cell cycle progression at the G1/S boundary (Serrano *et al*, 1993). Additional biological functions in processes such as cell senescence, invasive growth, cell spreading, apoptosis, anoikis and angiogenesis were subsequently reported. p16^INK4a^-deficient mice present with increased incidence of spontaneous and carcinogen-induced cancers (Kim and Sharpless, 2006). More recently p16^INK4a^ was associated with the clonal evolution of epidermal cells such that inactivation of p16 maintained epidermal cells in the stem cell compartment (Maurelli *et al*, 2006). In extension of these findings, knockout models have unveiled an age-dependent, tissue-specific function of p16 in limiting stem cell renewal (Kim and Sharpless, 2006).

A central role of p16 in pancreatic carcinogenesis was firmly established from the analysis of clinical specimens and corroborated by subsequent genetic studies in mice, as tissue-specific oncogenic activation of K-ras in Ink4a/ARF^−/−^ or Ink4A^−/−^/ARF ^+/+^ mice, but not in wild-type mice resulted in pancreatic ductal adenocarcinoma (Aguirre *et al*, 2003; Bardeesy *et al*, 2006). Also, tumour formation in the pancreas of p53^−/−^ mice overexpressing TGF-α was accompanied by deletion of the INK4a locus (Wagner *et al*, 2001). Owing to the tumour suppressor function, p16 reconstitution represents an appealing therapeutic approach. Accordingly, the biological effects following reconstituting p16 function *in vitro* have been extensively characterised (Rocco and Sidransky, 2001) and p16 was shown to inhibit growth of subcutaneous xenografts (Spillare *et al*, 1996; Rocco *et al*, 1998). However, little is known about the consequences of p16 re-expression for more complex aspects of tumour biology such as vascularisation and metastatic spread.

This may reflect on the experimental limitations of the subcutaneous approach, which fails to provide a representative microenvironment (Killion *et al*, 1998). Therefore, we aimed to study the effects of p16 in the more physiologic microenvironment of an orthotopic xenograft model. We furthermore, were concerned about epigenetic changes that might accompany the selection of clones with stable overexpression of p16. Therefore, an inducible expression system seemed mandatory.

In consequence, we combined an inducible expression system in pancreatic cancer cells with an orthotopic tumour model to study the effects of p16 reconstitution on tumour formation, growth, invasion, angiogenesis and metastasis in a representative *in vivo* model.

## Materials and methods

### Cell culture

MiaPaCa-2 cells (ATCC, Manassas, VA, USA) were grown as subconfluent monolayers in DMEM (Invitrogen, Berlin, Germany) containing 10% FCS, 2 mmol l^−1^ glutamine and 100 U ml^−1^ penicillin/streptomycin (Biochrom, Berlin, Germany) at 37°C in 95% air and 5% CO_2_.

### Cloning and generation of the tetracycline inducible cell line MiaPaCa-2-TREx-p16

The components of the TREx system (Invitrogen, Berlin, Germany) include the regulatory plasmid (pcDNA6/TR), the inducible plasmid (pcDNA4/TO) and a luciferase encoding reporter plasmid (pcDNA4/TO-Luc). MiaPaCa-2 cells were transfected (Effectene, Quiagen, Hilden, Germany) with pcDNA6/TR and stable clones (MiaPaCa-2-TREx) were selected by blasticidin (5 *μ*g ml^−1^). TetR promoter activation was achieved with 1 *μ*g ml^−1^ Doxycycline (Sigma Chemical Co., Deisenhofen, Germany) and responsiveness was evaluated by transient transfection with pcDNA4/TO-Luc followed by a luciferase assay (Promega, Mannheim, Germany). The full length cDNA of p16 was subcloned into pcDNA4/TO using restriction enzymes *Hin*dIII and *Kpn*I (Invitrogen, Berlin, Germany), resulting in pcDNA4/TO-p16. MiaPaCa-2-TREx-p16 cells were obtained through transfection of pcDNA4/TO-p16 into the MiaPaCa-2-TREx cells and selection with zeozin (0.5 mg ml^−1^)/blasticidin (5 *μ*g ml^−1^) (Invitrogen, Berlin, Germany).

### Western blotting

Cell lysates were prepared and blotted as described (Plath *et al*, 2000). Membranes were blocked and reacted overnight at 4°C with monoclonal antibodies (anti-p16^INK4a^ (1 : 1000) BD Pharmingen, Heidelberg, Germany), anti-pRB (1 : 1000) BD Pharmingen), anti-VEGF-C (Zytomed, Berlin, Germany) or VEGF-D (von Marschall *et al*, 2005), followed by appropriate secondary antibodies. Immunoreactive bands were visualised using an enhanced luminol reagent (Perkin-Elmer Life Science, Boston, USA).

### Cell proliferation (AlamarBlue assay)

Alamar Blue dye was purchased from Biosource International Inc. (Camarillo, CA, USA) and the manufacturer's instructions were followed to complete the assay (Ahmed *et al*, 1994; Nakayama *et al*, 1997).

### Anchorage-independent growth

For evaluation of anchorage-independent growth, 10^3^ cells were diluted in 1 ml methylcellulose/agar mixture resolved in Iscove's medium (Invitrogen, Berlin, Germany) supplemented with 27% fetal bovine serum (HyClone, Utah, USA). Cells were plated in triplicate on 3-cm dishes and incubated for 10 days. Colonies containing >20 cells were counted with an inverted light microscope.

### Animals

Female NMRI nude mice were purchased from Taconic M&B, Denmark. The studies were performed in accordance with a protocol approved by the regional animal research committee the ‘Landesamt für Gesundheit und Soziales Berlin Nr. A0136/01’. The *in vivo* procedures were in compliance with the UKCCCR guidelines.

### Orthotopic implantation

Orthotopic implantation was carried out when mice were about 7–8 weeks old. General anaesthesia was performed by intraperitoneal injection of a mixture of ketamine (100 mg kg^−1^) and xylazine (20 mg kg^−1^). In 2 × 12 animals, 1 × 10^6^ MiaPaca-TREx-p16 cells were injected into the head of the pancreas to visibly infiltrate the pancreatic tissue (Alves *et al*, 2001). In two mice the implantation failed due to a loss of cells into the abdomen during the procedure. Therefore the mice have to be killed. In successful implanted animals (*n*=22) p16 induction was started one day after tumour cell inoculation by adding doxycycline (2 mg ml^−1^) plus 2% sucrose to the drinking water (*n*=11 mice), whereas control animals received 2% sucrose alone (*n*=11 mice). All animals tolerated the procedure well. After implantation, mice were inspected daily for body weight loss, general condition, and tumour formation in the peritoneal cavity (abdominal distension). At the time of killing, primary tumours were removed and weighted. Tumour volume was calculated using the formula length × width × depth × *π*/6. Mesenterial and liver hilus lymph nodes were routinely collected for further examination.

### Immunohistochemistry

Primary tumours and lymph nodes were analysed by standard immunoperoxidase staining procedure. Briefly, 7 *μ*m thick cryosections were fixed in 4% paraformaldehyde/PBS, pH 7.0, for 20 min at room temperature (RT), treated with 0.3% H_2_O_2_ for 10 min and incubated overnight at 4°C with anti-human p16^INK4a^ (NEOMarkers, CA, USA) or 1 h at RT with antibodies to mouse LYVE-1 (Relia Tech, Braunschweig, Germany), mouse CD31 (BD Pharmingen, Heidelberg, Germany) or human pan-cytokeratin-HRP (Santa Cruz Biotechnology, CA, USA) (1 : 50) followed by incubation with a biotinylated secondary antibody. Immunoreactivity was detected with an avidin–biotin complex method (Vectastain Elite ABC kit) and AEC substrate. Sections were counterstained with hematoxylin.

### Angiogenesis and lymphangiogenesis

For quantitation of microvessel density (MVD) and lymphatic vessel density, respectively, the average numbers of CD31 and LYVE-1 positive vessels from three areas of maximal vascular density (vascular hotspots) were counted (Weidner, 1995).

### Statistical analysis

Data were analysed by *t*-test and Fishers exact test using Graph Pad statistical software (GraphPad Software Inc., San diego, CA, USA). Differences were considered significant at *P*<0.05.

## Results

### Generation of MiaPaCa-2 pancreatic cancer cells with doxycycline-inducible expression of p16

To obtain inducible p16-expression in pancreatic cancer cells *in vivo*, a modified tetracycline-inducible expression system (Gossen *et al*, 1995; Yao *et al*, 1998) was chosen, which permits *in vivo* gene induction by adding tetracycline to the drinking water.

**Figure 1:**
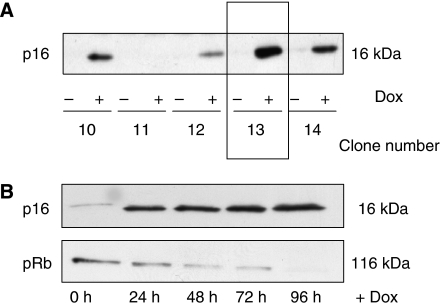
Generation MiaPaCa-2-TREx–p16 cells with doxycyclin-inducible expression of p16. (**A**) The plasmids pcDNA6/TR and pcDNA4/TO-p16 were sequentially transfected into p16-deficient MiaPaCa-2 cells and clones with inducible expression of p16 were selected (MiaPaca-2-TREx-p16). For each of the zeozine-resistant MiaPaCa-2-TREx-p16 clone, cells were grown for 48 h in the presence (+Dox) or absence (−Dox) of 1 *μ*g ml^−1^ doxycycline. The expression of p16 protein was analysed by western blotting. The clones with the highest p16 induction and lowest basal expression were further analysed, for example clone 13. (**B**) Time course of doxycycline-induced p16 expression. Cells were cultured for 96 h in the presence or absence of doxycycline and each 24 h, lysates were prepared for western blot analysis of p16 (upper panel) and pRB (lower panel). Equal amounts of protein (20 *μ*g) were separated on sodium dodecyl sulphate polyacrylamide gel electrophoresis.

Initially, we established MiaPaCa-2 cells with stable integration of pcDNA6/TR, which encodes the tet-repressor. Clones with abundant expression were subsequently identified in transient cotransfections with a tet-repressor-regulated luciferase reporter construct (data not shown). A clone with approximately 10-fold induction (clone MiaPaCa-TREx-10) was identified and utilised in a second transfection to introduce pcDNA4/TO-p16, which encodes p16 under the control of a tet-responsive promoter, such that tetracycline or doxycycline induce p16 expression. Western Blot analysis of multiple independent stable transfected clones revealed variable responses to doxycycline, ranging from no effect (clone number 11) to high expression of p16 associated with varying degrees of promoter leakage (Figure 1A, clone 13, 14). We chose two clones (number 13 and 16, not shown) for further studies, as these clones provided pronounced induction of p16 expression with minimal ‘leakage’ of basal p16 expression in the absence of doxycycline. The latter was of particular importance to ascertain that cells maintained the characteristics of parental MiaPaca-2 cells under control conditions. More detailed analyses revealed that p16-induction was rapid in onset, resulting in substantial amount of p16 at 24 h followed by a modest further increase up to 96 h. These analyses also confirmed the negligible background expression in the absence of Dox treatment.

Because p16 has been shown to suppress pRB expression *in vitro*, we hypothesised that pRB expression could provide a simple indicator for the functional integrity of the p16 molecule expressed from the Dox-regulated vector system. Indeed, induction of p16 in MiaPaCa-2 TREx-p16 cells reduced pRB content, indicating that a functionally intact p16 molecule was induced (Figure 1B).

### Effects of doxycycline-induced p16 expression on growth of MiaPaCa-2-TREx cells *in vitro*

**Figure 2:**
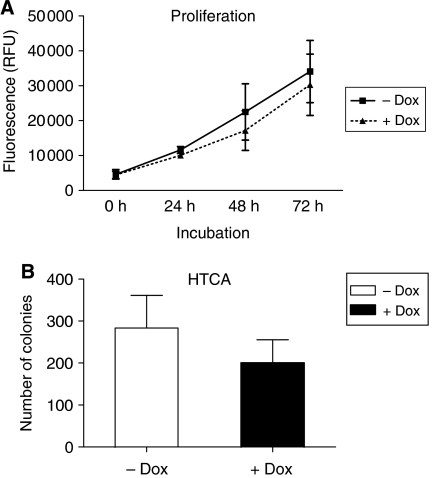
Effects of doxycycline-induced p16 expression on growth of MiaPaCa-2-TREx cells *in vitro*. (**A**) Time-dependent proliferation of MiaPaCa-2-TREx-p16 cells in the presence and absence of Dox as determined based on Alamar Blue assay. Every 24 h alamar blue dye was applied to the media of the cells and fluorescence was determined as an indirect measurement of cell numbers. (**B**) Effects of p16 expression on anchorage-independent growth of MiaPaCa-2-TREx-p16/13 cells. Anchorage-independent growth was evaluated based on soft agar colony formation. 10^3^ cells were seeded out in a methylcellulose/agar mixture and incubated in the presence (+Dox) or absence (−Dox) of doxycycline for 10 days. Vital colonies were counted. Values shown are the mean±s.e.m. for each group (+/−Dox) of three independent experiments.

Effects of p16 on proliferation of MiaPaCa-2 cells *in vitro* were studied next. A time-dependent increase in cell numbers was observed both in the presence and absence of Dox. Although a trend towards a reduction of cell numbers was observed in Dox-treated cultures, this effect did not reach statistical significance (Figure 2A).

In our previous studies, p16 restitution in Capan-1 cells abrogated tumorigenicity *in vivo* (Plath *et al*, 2000), which was reflected by a severe reduction of colony formation *in vitro*. We therefore examined the impact of p16 induction on colony formation of MiaPaCa-2 cells and observed a ∼30% decrease following addition of Dox (Figure 2B). Comparable results were obtained with MiaPaCa-2-TREx-p16 clone no. 16 (data not shown).

Taken together, inducible p16 expression elicited minor direct growth inhibitory effects on MiaPaCa-2 cells *in vitro*. Thus, this cell model seemed ideally suited to provide information about the context driven effects of p16 on tumour growth.

### Induction of p16 inhibits primary tumour growth of orthotopic MiaPaCa-2 xenografts

As we intended to study the inducible MiaPaCa-2-TREx-p16 cell system in the orthotopic context, we initially had to confirm the feasibility of this approach. Indeed, MiaPaca-2-TREx gave rise to extensive local tumour growth with invasion of adjacent organs exactly as parental MiaPaca-2 cells. Furthermore, Dox treatment of mice carrying MiaPaca-2-TREx cells was well tolerated and did not significantly reduce primary tumour weight, suggesting that Dox treatment *per se* would not confound growth regulatory effects of p16 induction (data not shown).

**Figure 3:**
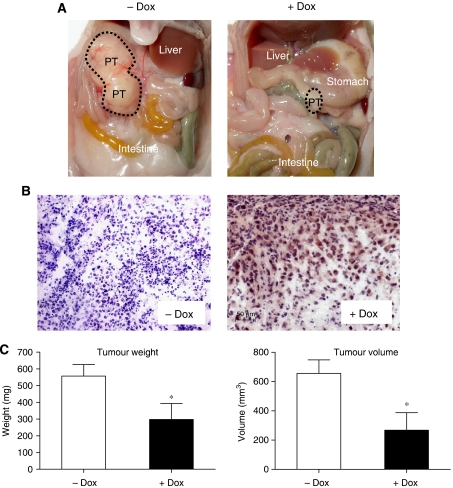
Induction of p16 expression reduced primary tumour growth in orthotopic MiaPaCa-2 pancreatic carcinomas. Pancreatic MiaPaCa-2-TREx-p16 cells were grown orthotopically in the pancreas of mice treated with or without Dox as indicated. At the end of the treatment (8 weeks) tumour volumes and weights were calculated. (**A**) Open situ of representative mice from the control (−Dox) and treatment group (+Dox). (**B**) Confirmation of p16 induction *in vivo*. Primary tumours were analysed for p16 expression by immunohistochemistry using an antibody against p16. An example of p16 expression in a small tumour in the treatment group is shown on the right panel, with detection of p16 protein in the nucleus and in the cytoplasm of tumour cells. In contrast, no signal was obtained in control tumours (−Dox) (images at × 20 magnification). (**C**) Summary of primary tumour weights (left) and volumes (right) from Dox-treated animals. Data represent mean±s.e.m. for each group. ^*^P<0.05.

We then proceeded to induce p16 expression in tumour bearing mice as described in material and methods. After 8 weeks mice were killed and primary tumours were removed to determine volume and weight. Representative pictures of the abdominal situs in mice from control (−Dox) and treatment groups (+Dox) at the time of autopsy are presented in Figure 3A. Mean tumour weight and volume of primary tumours were lower in treated animals, but a conspicuous variation of values in the treatment group precluded statistical significance. We hypothesised that this variability might reflect differences in the level of p16 induction and accordingly examined p16 expression by immunohistochemistry. Successful *in vivo* induction was evident in 6 out of 11 mice with MiaPaCa-2-TREx-p16 orthotopic tumours (Figure 3B). p16 was absent in 8 out of 11 mice from the control group, but a weak signal was obtained in the remaining three control tumours, possibly due to promoter leakage. Based on these results, we excluded tumours of animals without immunohistological corroboration of p16 expression or absence from the analysis. When weight and volume from the tumours with confirmed re-expression of p16 were compared to values from controls, a significant reduction by 60 and 52%, respectively, was noted (Figure 3C). Furthermore, several control tumours, but none with p16 induction invaded into surrounding tissues as stomach and duodenum (data not shown). Altogether, re-expression of p16 in pancreatic cancer cells substantially reduced orthotopic tumour growth *in vivo* and thus surpassed the growth inhibitory effects *in vitro*.

### Induction of p16 expression reduced angiogenesis in orthotopic MiaPaCa-2 pancreatic carcinoma

**Figure 4:**
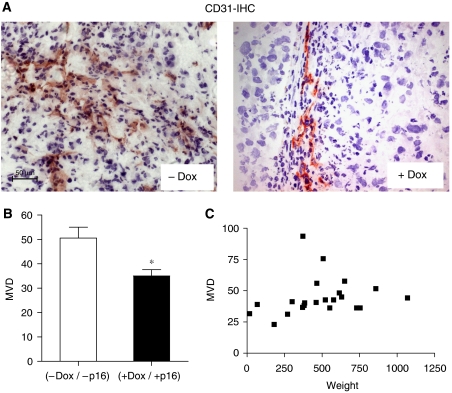
Induction of p16 expression reduced angiogenesis in orthotopic MiaPaCa-2 pancreatic carcinomas. Cryosections of the tumours from control (−Dox) and treatment group (+Dox) were stained with the endothelial marker CD31. To determine microvessel density (MVD) CD31-expressing vessels were quantitated from hotspot areas, as described in Materials and methods. (**A**) Shown are representative CD31 stainings from a (−Dox) and a (+Dox) tumour (**B**) and quantitative evaluation of the results, ^*^P<0.05. (images at × 20 magnification) (**C**) Relationship of MVD and tumour weight. MVD values from both groups (+/−Dox) were plotted against the respective tumour weight to assess a potential correlation of both parameters. The distribution shows no correlation.

Growth inhibition may have resulted from direct antiproliferative or proapoptotic effects of p16 in the tumour cells or from indirect mechanisms. As vascularisation is a prerequisite for tumour growth, we examined whether p16 affected angiogenesis. Cryosections of the primary tumours were stained with CD31 (Figure 4A) to detect endothelial cells and average MVD was determined. MVD values in the control group ranged from 38 to 76, indicating that the MiaPaca-2 tumours were well vascularised. Induction of p16 significantly reduced the MVD (35 *vs* 51, *P*=0.0123, Figure 4B). It is noteworthy that tumour size *per se* did not correlate with MVD (Figure 4C), indicating that reduced MVD observed in the treatment group likely reflected a specific biological effect of p16 restitution.

### Induction of p16 expression reduced lymphangiogenesis in orthotopic MiaPaCa-2 pancreatic carcinoma

**Figure 5:**
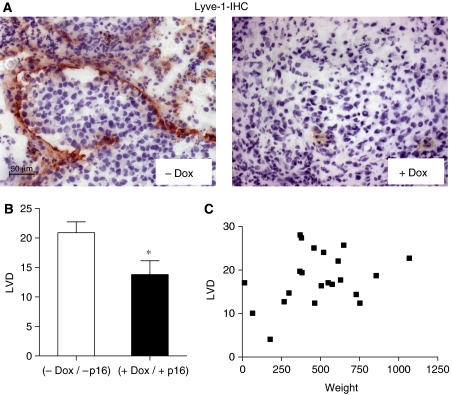
Induction of p16 expression reduced lymphangiogenesis in orthotopic MiaPaCa-2 pancreatic carcinomas. Cryosections of the tumours were stained with Lyve-1, an endothelial cell marker, which specifically detects lymphatic endothelia. (**A**) Illustration of Lyve-1 immunohistochemical detection in representative tumours from control (−Dox) and treatment group (+Dox) (images at × 20 magnification). (**B**) Quantitative evaluation of lymphatic vessel density (LVD) for each group (+/−Dox). ^*^P<0.05. (**C**) Missing correlation of LVD and tumour weight.

Owing to the reduction in MVD apparent from CD 31 staining could result from an inhibition of either hem- or lymphangiogenesis, the latter was separately determined based on the immunostaining with LYVE-1 (Figure 5A), an antibody that recognises the hyaloronan receptor-1 of lymphatic vessels. Tumours with confirmed p16 induction (+Dox) contained less Lyve-1 positive lymphatic vessels than control tumours (33% reduction, *P*=0.0299, Figure 5B). Similar to MVD, LVD did not correlate with tumour size (Figure 5C). Thus, re-expression of p16 inhibited lymphangiogenesis.

### Induction of p16 expression reduced metastasis in orthotopic MiaPaCa-2 pancreatic carcinoma

**Figure 6:**
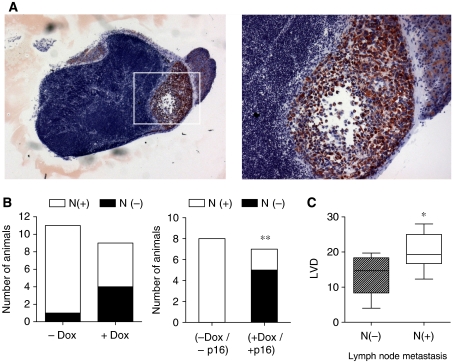
Induction of p16 expression inhibits lymphatic spread of MiaPaCa-2 cells to the liver hilus lymph nodes. Metastatic spread of MiaPaCa-2-TREx-p16 cells was evaluated based on inspection of lung and abdominal organs at the time of autopsy and microscopic assessment of lymph node metastasis. (**A**) Representative picture of a lymph node with cytokeratin-positive human tumour cells (stained red) surrounded by lymphocytes (images at × 10 magnification and × 20 (image inset). (**B**) Quantitative analysis of lymph node metastasis. Data represent the number of animals with presence of lymph node metastasis in the liver hilus lymph nodes (left: comparison between treatment (+Dox) and control group (−Dox). Right: subgroup analysis including only those animals with histologically confirmed expression or absence of p16 protein (−Dox/p16 *vs* +Dox/+p16) (^**^P<0.05) whereas in (**C**) the correlation between LVD and lymph node metastasis is shown (^*^P<0.05).

As tumour size, tumour angiogenesis and lymphangiogenesis are important determinants of metastatic tumour progression, metastasis formation of orthotopic MiaPaCa-2 tumours was examined. On macroscopic inspection the abdominal organs and lungs of either treatment or control group were tumour-free. At the microscopic level however (Figure 6A), human MiaPaCa-2-TREx-p16 cells were detected in liver hilus lymph nodes of 10 out of 11 mice in the control group (91%), using an anti-human pan-cytokeratin antibody (Figure 6A and B). In contrast, hilar lymph node metastases had occurred in only one of the tumours with re-expression of p16 (Figure 6B). Mesenterial lymph nodes had not been invaded by metastatic cells in any of the mice (data not shown).

As lymphatic spread was the prevalent mode of metastasis in MiaPaca-2-TREx-p16 tumours, we compared the LVDs in tumours from mice with (N+) or without lymph node infiltration (N−) (Figure 6C). A significant difference in LVDs was observed between groups, suggesting that reduced lymphvascularisation in tumours with p16 expression had impaired their capacity for metastatic spread. In contrast, no difference was found with respect to hemangiogenesis as calculated from the difference of MVD and LVD (data not shown).

## Discussion

Functional inactivation of the tumour suppressor p16 marks a key event in pancreatic carcinogenesis. Conversely, reconstitution of the biological action of p16 provides an attractive therapeutic approach. Here, we addressed the consequences of p16 re-expression in MiaPaca-2 pancreatic cancer cells *in vivo* in the orthotopic tissue context. Consistent with previous work in subcutaneous xenograft models (Spillare *et al*, 1996; Harada *et al*, 1999; Hosotani *et al*, 2002), we found p16 capable of reducing primary tumour growth. In addition, p16 restitution resulted in a marked reduction of tumour angiogenesis, largely accounted for by a p16-dependent inhibition of lymphangiogenesis. In excellent agreement with the antilymphangiogenic effect, re-expression of p16 prevented lymph node metastases of MiaPaca-2 pancreatic cancer cells. To our knowledge, this is the first report that experimentally links p16 to the process of lymphangiogenesis.

The restitution of p16 function remains an attractive therapeutic approach and a focus of drug development (Senderowicz and Sausville, 2000; Hosotani *et al*, 2002; Kondo *et al*, 2004).

So far, little is known about the consequences of p16 re-expression for more complex aspects of organ-specific tumour growth, which we focussed on in the current study. To this end, we combined a tetracycline-inducible expression system with an orthotopic approach, which allowed to overcome several potential obstacles.

The inducible system permitted selection, expansion, and continuous culture of stable clones, while avoiding the selection pressure expected from overexpression of a tumour suppressor. Upon addition of Dox, functionally relevant expression of p16 in MiaPaca-2-TREx-p16 was reproducibly obtained as judged from the ability to downmodulate pRB (Fang *et al*, 1998) and to reduce colony formation in soft agar (Plath *et al*, 2000), but did not block proliferation of MiaPaca-2 cells. Thus, the system seemed well suited for further *in vivo* use.

For several reasons, the orthotopic approach seemed of particular benefit: First, primary tumours were allowed to form and progress within their correct microenvironment. Thus, tissue-specific interactions between tumour cells and the stromal and vascular compartments, which are increasingly recognised as major determinants of the tumour phenotype, were maintained (Blouw *et al*, 2003; Chu *et al*, 2007). Second, orthotopic pancreatic tumours metastasised using the clinically relevant route of lymphatic metastasis and thereby offered information that is unavailable from subcutaneous xenografts. Third, the orthotopic approach in nude mice allowed to utilise human carcinoma cells, which are expected to reflect the characteristic molecular makeup of the human disease. Thus, the orthotopic model offers the potential to obtain clinically relevant data on the effects of p16 restitution in pancreatic cancer (Killion *et al*, 1998).

Although a successful combination of the inducible system with the orthotopic approach was feasible, almost one-third of the Dox-treated tumours did not express p16 at the time of autopsy. This may result from insufficient Dox delivery to the tumour cells or from selection pressure to inactivate the tumour suppressor. As these tumours may have been subjected to p16 expression for an uncertain period of time, they had to be omitted from most analyses. From the analyses of the remaining tumours with confirmed p16 expression, several insights were obtained.

MiaPaca-2-TREx-p16 cells formed well vascularised orthotopic primary tumours. This angiogenic phenotype was not correlated to tumour size, suggesting it represented a characteristic feature of the tumour biology of MiaPaca-2 pancreatic tumours. Dense vascularisation was also confirmed with respect to lymph endothelia, which have become a focus of research due to their role in lymphatic metastasis (Stacker *et al*, 2002). Induction of p16 *in vivo* profoundly affected the angiogenic phenotype of MiaPaca-2-TREx-p16 tumours, resulting in decreased microvessel density and lymphatic vessel density.

The p16-mediated suppression of the angiogenic phenotype observed here is in excellent agreement with data obtained by inactivation of p16 in the MIN (Multiple Intestinal Neoplasia) mouse model of colon carcinogenesis (Gibson *et al*, 2003, 2005). Colonic tumours in MIN mice with additional p16 deletion revealed a strikingly increased vascularisation and slightly larger size as compared with p16-competent control animals. This effect was restricted to tumours in the large intestine, suggesting tissue-specific aspects in the regulation of angiogenesis by p16. Such tissue specific effects possibly result from differences in the composition of proangiogenic factors from tumour cells, stromal cells and hypoxia (Craft and Harris, 1994). In this context, p16 was shown to suppress VEGF-A expression *in vitro* (Harada *et al*, 1999) and loss of p16 correlated with VEGF-A overexpression in human oesophageal squamous cell carcinoma specimens (Takeuchi *et al*, 2004). However, p16 induction in MiaPaca-2-TREx-p16 cells *in vitro* did not affect VEGF-A protein levels in cells or supernatants (data not shown), suggesting p16-controlled angiogenesis through alternate mechanisms.

One key contribution to the overall reduction of MVD was the marked decrease of lympahtic vessel density. This experimental link between p16 and lymphangiogenesis provides a pathobiological concept for earlier clinical observations that associated the loss of p16 with lymph node metastasis in several types of carcinomas (Kawabuchi *et al*, 1999; Gessner *et al*, 2002). Unfortunately, we could not examine this relationship in pancreatic cancer due to the scarcity of specimens with functionally intact p16.

Published work on lymphangiogenesis in pancreatic cancer has identified VEGF-C and VEGF-D as important stimulators (Tang *et al*, 2001; von Marschall *et al*, 2005) prompting us to test the effects of p16 induction on the amount of VEGF-C or -D produced in MiaPaca-2-TREx-p16 cells *in vitro*. However, neither of these two VEGF family members were found regulated, suggesting that additional factors controlled lymphangiogenesis in MiaPaca-2-TREx-p16 tumours.

Irrespective of the mechanism whereby p16 reduced lymphangiogenesis our results strongly support the proposed sequence of p16 induction, reduction of lymphangiogenesis, and prevention of lymphatic metastasis in pancreatic cancer, as (i) p16 significantly inhibited lymph node metastasis, and (ii) mice with lymph node infiltration had significantly higher LVDs in their primary tumours. Given the importance of lymphatic metastasis for the clinical management of pancreatic cancer, our observations provide an important insight into the molecular events that determine this process. They furthermore suggest that p16 mimetic therapies provide benefits beyond the reduction of tumour cell proliferation and primary tumour burden.
